# A Comprehensive Study of the Effect of Lubricant in the Sizing Agent on the Properties of a Basalt Fiber and Epoxy Resin Composite Material

**DOI:** 10.3390/nano15110838

**Published:** 2025-05-30

**Authors:** Jiajun He, Chuan Lai, Junlan Li, Ning Yang, Bin Xie, Xiaolong Li, Yuanfang Deng, Like Zou

**Affiliations:** 1 School of Chemistry and Environmental Engineering, Sichuan University of Science and Engineering, Zigong 643000, China; hejiajun9826@163.com (J.H.); junlan314@foxmail.com (J.L.); 15281340435@163.com (N.Y.); xiebin@suse.edu.cn (B.X.); 2 Dazhou Key Laboratory of Advanced Technology for Fiber Materials, Sichuan University of Arts and Science, Dazhou 635000, China; 15183059113@163.com; 3 Dazhou Quality Technology Supervision Inspection and Testing Center, Dazhou 635000, China; lc860801@163.com

**Keywords:** sizing agent, lubricant, basalt fiber, reinforced polymer

## Abstract

Based on the formula for the sizing agent for basalt fiber, this paper presents a comprehensive study of the effects of lubricants on the properties of sizing agents, basalt fiber, and epoxy resin composite materials. Through testing and analysis of physical and chemical parameters, a new sizing agent with excellent performance was developed. The results demonstrated that the components and proportions of the lubricant significantly affected the physical and chemical parameters of the emulsion, as well as the mechanical properties of the basalt fibers and their epoxy resin composite materials. The lubricant with the combination ratio of 0.70% saturated fatty acid polyoxyethylene ester and 0.30% unsaturated fatty acid polyoxyethylene ester and imidazoline lubricant-I produced basalt fiber with the best mechanical properties. The single fiber tensile strength and yarn breaking strength increased by 18.42% and 12.5%. Furthermore, the lubricant with the combination ratio of 0.70% saturated fatty acid polyoxyethylene ester and 0.30% unsaturated fatty acid polyoxyethylene ester and imidazoline lubricant-III resulted in the best mechanical properties for Epoxy–BFRP composite materials. The tensile strength of the Epoxy–BFRP composite material increased by 13.2%, the tensile modulus increased by 45.2%, and the flexural strength increased by 12.0%.

## 1. Introduction

Basalt fiber (BF) is an inorganic non-metallic fiber created by melting natural basalt rocks at temperatures between 1400 and 1500 °C using a platinum–rhodium alloy spinner to draw the molten material into fibers. The chemical composition and structure of BF endow it with inherent properties such as natural purity, wear resistance, high-temperature oxidation resistance, corrosion resistance, and cost-effectiveness. Considered a “green strategic resource”, BF production does not generate “three wastes” (i.e., waste gas, wastewater, and waste residue) [1,2,3,4,5]. Due to its outstanding performance, BF has found widespread applications in industries such as aerospace, automotive, construction, and energy conservation [6].

Given the smooth and chemically inert nature of BF surfaces, surface treatment is essential during usage [7,8]. Typically, surface treatment involves applying a sizing agent during the spinning process. The coating formed by the sizing agent can repair surface defects on the fibers, effectively dispersing stress on the fiber surface and enhancing its mechanical properties [9]. In the case of BF-reinforced resin composites, the bond between BF and the resin directly influences the mechanical properties of the composite material. The sizing agent plays a crucial role in affecting the physical, chemical, and mechanical properties of both the fiber and the composite material to varying degrees [10,11,12,13,14,15].

Currently, film-forming agents, lubricants, and coupling agents constitute the three essential components in the majority of sizing agents. Additionally, various formulations of the sizing agent may incorporate additives such as wetting agents, antistatic agents, and pH regulators, among others. Apart from film-forming agents, lubricants hold the largest proportion and have a pronounced effect. They effectively reduce the frictional forces during the production process of BF raw silk, preventing fiber damage. Moreover, lubricants contribute to maintaining a smooth texture in the final BF product, reducing fuzziness and effectively decreasing the friction between fibers and resin, enhancing the infiltration efficiency of fibers and lowering the manufacturing costs of composite materials [16]. While some studies on the influence of film-forming agents and coupling agents on BF and its reinforced composite materials have been reported in recent years [5,17,18,19,20,21,22], the impact of lubricants on BF and composite materials is relatively limited. Therefore, the effect of lubricants on the properties of BF products and BF reinforced resin composites is of paramount significance to study.

Against this backdrop, this study aims to investigate the formulation of basalt fiber sizing agents, with a focus on the effects of lubricants in the sizing agents on the properties of basalt fibers and epoxy resin composites. The formulation of the sizing agents will be designed using a controlled-variable method, with the primary evaluation criteria being the physicochemical properties of the sizing agents and the mechanical properties of fibers and BF-reinforced resin composites, which will be used to assess the advantages and disadvantages of new lubricants. By improving the compatibility of lubricants with BF sizing agents, this study seeks to develop superior basalt fiber sizing agents, thereby enhancing the comprehensive performance of existing BF and its composites by at least 10%.

## 2. Experimental

### 2.1. Materials

Basalt fiber (BF), with an average diameter 16 ± 1 um and a linear density of 215~250 tex, was supplied by Sichuan Juyuan Basalt Fiber Co., Ltd. (Dazhou, China), and BF unidirectional fabric with a surface density of 300 g m^−2^, a thickness of 0.2 mm, and a width of 50 cm was subcontracted to Dazhou Zengmei Basalt Fiber Technology Co., Ltd. (Dazhou, China). Unsaturated fatty acid polyoxyethylene ester lubricant (L-1) and saturated fatty acid polyoxyethylene ester lubricant (L-2) were procured from Nantong Qianhe Chemical Co., Ltd. (Nantong, China). Epoxy resin film-forming agent E21 emulsion, modified epoxy auxiliary film-forming agent, and amide-based cationic lubricant (L-3) were provided by Sichuan Juyuan Basalt Fiber Co., Ltd. (Dazhou, China). Dioleyl Imidazoline Quaternary Ammonium Cationic Lubricant I (L-4) was purchased from WanHua Supply Chain Service Co., Ltd. (Guangzhou, China) non-ionic imidazoline-based lubricant II (L-5) and cationic imidazoline-based lubricant. III (L-6) were sourced from Xi’an Youji Composite Materials Co., Ltd. (Xi’an, China). Coupling agent KH570 was acquired from Jiangxi Chenguang New Materials Co., Ltd. (Jiujiang City, China). Epoxy resin SM828 was obtained from Jiangsu Sanmu Chemical Co., Ltd. (Yixing City, China). The curing agent polyamide resin 651 was procured from Yichun Junzheng New Materials Co., Ltd. (Yichun, China).

### 2.2. Sizing Agent Preparation

The original formulation of the sizing agent (A0) was sourced from Sichuan Juyuan Basalt Fiber Co., Ltd. It primarily consists of a film-forming agent emulsion, an auxiliary film-forming agent, a lubricant, and a silane coupling agent, with specific component contents of 7.0%, 1.6%, 1.3%, and 0.60%, respectively. In sizing agents for basalt fibers, lubricants are generally used in combinations of two or more types. Excessive lubricant content in the sizing agent hinders the adsorption of coupling agents on the fiber surface, thereby reducing the effectiveness of coupling agents. Conversely, insufficient content can cause fiber abrasion and filament shedding during fiber drawing production and subsequent textile processing, affecting fiber performance. The original formulation A0 contains three lubricant components (Lubricant-I, Lubricant-II, and Lubricant-III) at weight ratios of 0.30%, 0.70%, and 0.30%, respectively. This combination and ratio have demonstrated excellent wet and dry sliding properties in both the industrial fiber drawing production and fabric weaving processes. Therefore, in this study, while maintaining the total lubricant content at 1.3%, nine different BF sizing agent emulsions (A1~A9) were obtained by varying the lubricant components and their proportions. The specific components and contents are listed in Table 1.

As shown in Table 1, the lubricants used in this study consist of two major categories totaling six types: non-ionic lubricants (including unsaturated fatty acid polyoxyethylene ester (L-1), saturated fatty acid polyoxyethylene ester (L-2), and alkyl imidazoline (L-5)) and cationic lubricants (including amide-based cationic (L-3), dioleic quaternary ammonium salt imidazoline cationic (L-4), and non-quaternary ammonium salt imidazoline cationic (L-6)). Non-ionic lubricants (L-1, L-2, L-5) achieve adsorption through hydrogen bonding between polar groups (polyoxyethylene chains/imidazoline rings) and hydroxyl groups on the fiber surface, as well as van der Waals interactions between hydrophobic chains and non-polar regions of the fiber. This adsorption behavior is influenced by temperature and the structure of hydrophobic chains (saturated/unsaturated). Cationic lubricants (L-3, L-4, L-6) carrying positive charges adsorb onto the negatively charged fiber surface primarily through electrostatic attraction (primary mechanism) supplemented by hydrophobic interactions (secondary mechanism). This results in a dense adsorption layer with significant charge effects.

**Figure 1:**
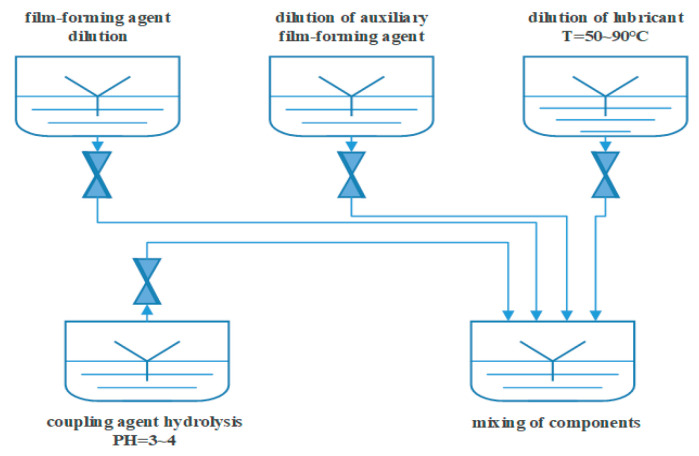
Preparation of the sizing agent emulsion.

The preparation process contains five steps, which are coupling agent hydrolysis, film-forming agent dilution, dilution of the auxiliary film-forming agent, dilution of the lubricant, and mixing of the components, as Figure 1 shows. Coupling agent hydrolysis: Take deionized water at 30 times the amount of coupling agent and add the glacial acetic acid dropwise to adjust the pH to 3~5. Under stirring conditions, add the coupling agent to the deionized water with the adjusted pH until the solution is clear. Film-forming agent dilution: According to the formulation ratio of the film-forming agent, take 1.5~2 times the amount of the film-forming agent in deionized water and dilute to obtain a film-forming agent emulsion in reserve. Dilution of the auxiliary film-forming agent: Based on the formulation ratio of auxiliary film-forming agent, take 3~5 times the amount of the auxiliary film-forming agent in deionized water and dilute in reserve. Dilution of the lubricant: Dilute 10~15 times with deionized water at 50~90 °C. Mixing of the components: Pour the diluted film-forming agent into the container and add the diluted auxiliary film-forming agent, lubricant, and coupling agent. We mixed the solution system with rapid stirring for 1.5 h, then homogenized for 5 min using a homogenizer to obtain the sizing agent emulsion.

### 2.3. Epoxy–Basalt Fiber Reinforced Polymer Preparation

**Figure 2:**
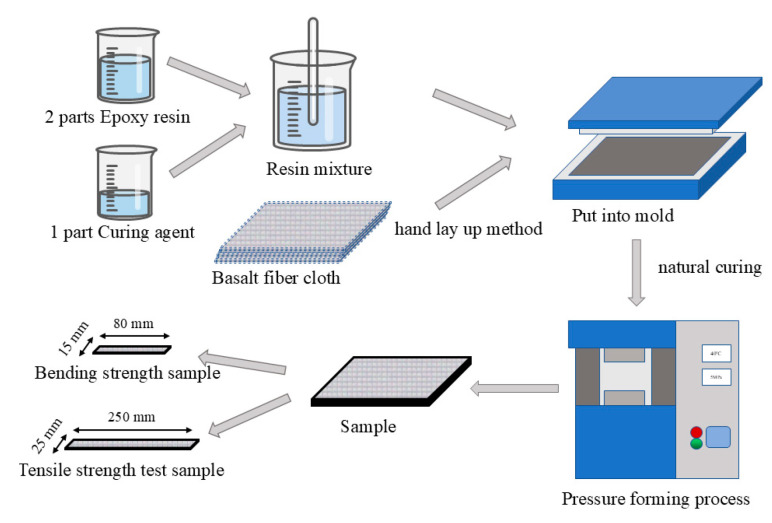
Composite material fabrication.

**Figure 3:**
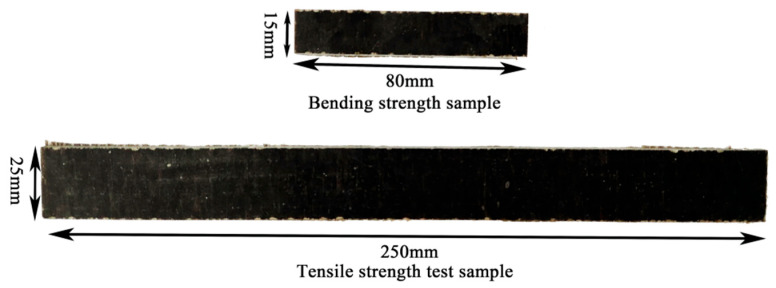
Testing specimens of composite materials.

The preparation of epoxy–basalt fiber reinforced polymer (Epoxy–BFRP) is illustrated in Figure 2. The matrix resin was prepared by mixing epoxy resin (SM828) and curing agent (Polyamide resin 651) at a mass ratio of 2:1. After thorough blending, the resulting mixture was evenly applied to the surface of the treated fiber cloth (360·360 mm). The fiber cloth was arranged in the mold using a 0° stacking method. Once the resin naturally solidified to a gel state, the assembly was placed on a flat vulcanization machine with the temperature and pressure controlled at 40 °C and 5 MPa, respectively, and was subjected to continuous pressure for 90 min. Subsequently, the obtained samples were removed and left to naturally cure. After cutting them into standard specimens (4·25·250 mm and 4·15·80 mm), they were used for subsequent testing. The Epoxy–BFRP specimens are depicted in Figure 3.

### 2.4. Characterization and Testing

#### 2.4.1. Evaluation of the Physical and Chemical Properties of the Sizing Agent

We utilized a centrifuge of type 80-1 (Shenglan Instrument Manufacturing Co., Ltd., Changzhou, China) and subjected the emulsion to centrifugal treatment at 3000 RPM for 30 min while watching for the presence of sedimentation or layering phenomena. A Brttersize3000Plus laser/image particle size analyzer (Dandong Bettersize Instruments Ltd., Dandong, China) was employed for the particle size distribution test. The testing range spanned 0.01~2000 μm, with a substance refractive index of 1.52 and an absorption rate of 0.1. Deionized water was utilized as the dispersing agent with a medium refractive index of 1.333. Viscosity analysis was conducted using the NDJ-8S digital viscometer (Shanghai Shangpu Instrument & Equipment Co., Ltd., Shanghai, China) at a rotational speed of 60 RPM; the Theta Flex optical contact angle meter (Biolin Scientific, Gothenburg, Sweden) was utilized with the pendant drop method; and the weight loss method was used to determine the solid content. Clean surface dishes without and with emulsion solutions were placed in a drying oven at 105 °C for 2 h. Based on this method, the solid content (*X*, %) was obtained by Equation (1), where *m* is the mass of the blank drying dish. *m_1_* and *m_2_* are the masses of the drying dish with the emulsion solution before and after drying. At least three closer results were considered for different testing, and their average values have been reported.

(1) $$X=\frac{m_{2}-m}{m_{1}-m}\times 100\%$$

#### 2.4.2. BF Performance Evaluation

**Figure 4:**
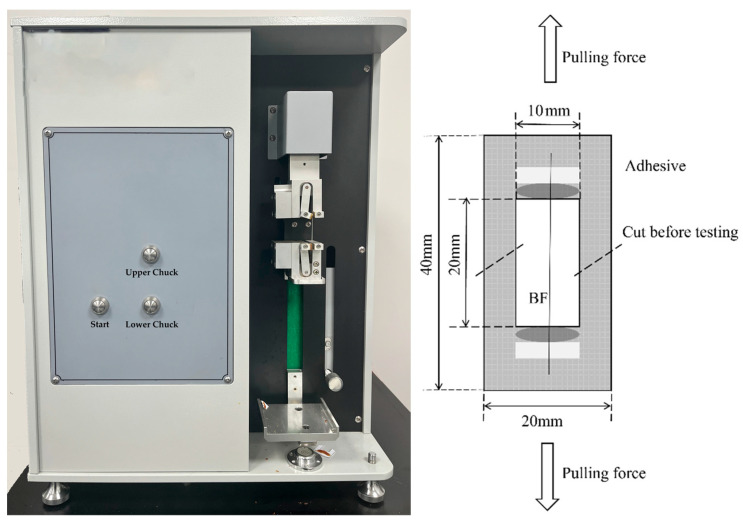
The YG004 electronic single fiber strength tester and a schematic diagram of the single fiber tensile strength test specimen.

Fiber linear density and diameter were determined according to GB/T 7690.1-2013 [23]. Moisture content was measured following the guidelines of GB/T 9914.1-2013 [24], and combustible content was assessed through GB/T 9914.2-2013 [25]. The single fiber tensile strength was determined using the YG004 electronic single fiber strength tester (Changzhou No.1 Textile Equipment Co., Ltd., Changzhou, China) in accordance with the requirements of DB51/T 2321-2017 [26], and a schematic diagram of the sample testing is shown in Figure 4. Yarn breaking strength was measured using the UTM6503 electronic universal testing machine (Shenzhen Sansi Zongheng Technology Co., Ltd., Shenzhen, China) according to the GB/T 7690.3-2013 standard [27].

#### 2.4.3. Epoxy–BFRP Composite Material Performance Evaluation

Tensile and flexural performance testing of Epoxy–BFRP composite materials was conducted in accordance with the standards GB/T 1447-2005 and GB/T 1449-2005 [28,29]. The tensile specimen dimensions were 4·25·250 mm with a loading rate of 5 mm min^−1^ applied across the gauge length. The flexural specimen dimensions were 4 15 80 mm with a loading speed of 5 mm min^−1^, as illustrated in Figure 3. The surface morphology of the composite material’s flexural failure was observed using a Sigma300 scanning electron microscope (SEM; Carl Zeiss (Shanghai) Management Co., Ltd., Shanghai, China ).

## 3. Results and Discussion

### 3.1. Influence of Lubricants on the Physical and Chemical Properties of the Sizing Agent

#### 3.1.1. Emulsion Stability

**Figure 5:**
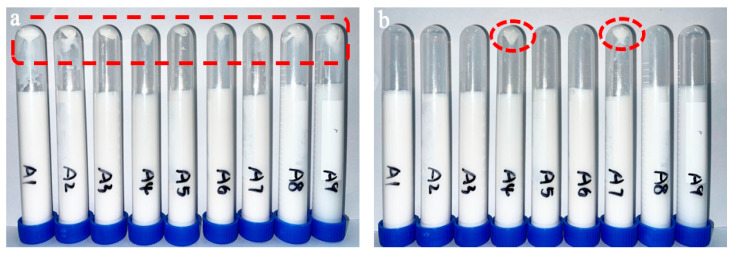
Centrifugal sedimentation treatment of sizing agent emulsions A1~A9: Sizing agent emulsion after centrifugation (**a**), sizing agent emulsion after shaking and sonication following centrifugation (**b**).

Lubricants with emulsifying properties can reduce the surface tension between droplets, promote their uniform dispersion in the continuous phase, and enhance emulsion stability. Centrifugal sedimentation was used to analyze the emulsion stability of nine BF sizing agent formulations (A1–A9), listed in Table 1, with specific results shown in Figure 5. After centrifugal sedimentation, the sizing agent emulsions of A1–A9 exhibited minimal sedimentation without obvious stratification (Figure 5a). For the small amount of sediment observed, shaking or sonication effectively redispersed it, except for formulations A4 and A7, where sedimentation persisted (Figure 5b). Among the nine formulations, A4 and A7 were classified as unstable, indicating poor emulsification and dispersion. In contrast, the remaining seven formulations showed negligible sedimentation due to minor density differences between phases, with no coalescence of emulsion particles. Under external force, the sediment redispersed, and the emulsion system returned to its original state, confirming no demulsification occurred.

The poor emulsion stability of A4 and A7 stems from the dual incompatibility of hydrophobic chain lengths and polar group interaction modes between the two non-ionic lubricants (L-5 and L-2) used in these formulations. L-5 (an imidazoline-based lubricant) features a short alkyl chain and a rigid imidazoline ring that adsorbs in a tilted/lying flat conformation, forming only single-point hydrogen bond-dipole interactions with water through ring nitrogen atoms. L-2 (a saturated fatty acid polyoxyethylene ester), however, has long saturated stearic acid chains that arrange vertically and densely at the oil–water interface, establishing a hydrogen bond network with water via polyethoxy groups. When mixed, L-5 and L-2 exhibit incompatible adsorption morphologies (tilted vs. vertical) and conflicting polar group interactions (single-point vs. network-forming), which disrupt the continuity of the overall adsorption film. Thus, emulsions A4 and A7, which contain both L-5 and L-2, are prone to demulsification due to these structural incompatibilities.

#### 3.1.2. Emulsion Size and Distribution

**Figure 6:**
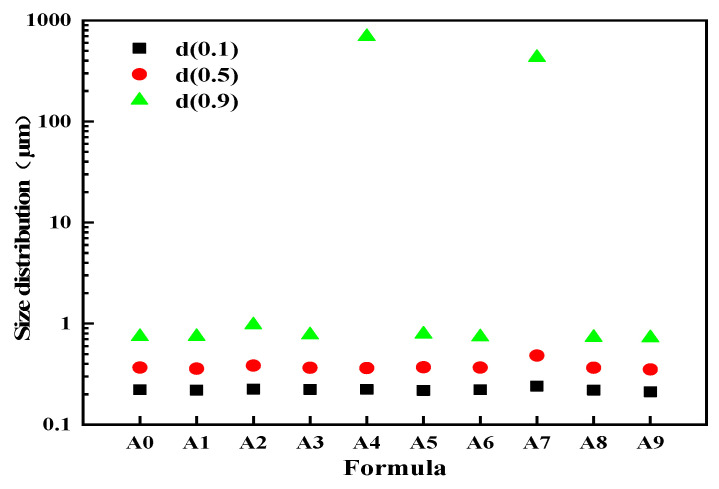
Size and distribution of sizing agent emulsion.

The results of the particle size and distribution testing for A0-A9 sizing agent emulsions are shown in Figure 6. From this figure, it is evident that when the ratio of lubricant components in the sizing agent varies, there are significant differences in emulsion particle size and distribution. The d (0.1) and d (0.5) values indicate a nanoscale average particle distribution in the sizing agent, while the data for d (0.9) show larger variability. The particle size of the A4 sizing agent emulsion is 690.0 µm, and the particle size of the A7 sizing agent is 428.8 µm. An analysis of the operating process revealed that one possible cause of this phenomenon was the mismatch of lubricant components in A4 and A7, making it challenging to stably disperse each component in the sizing agent system. Further evidence from the centrifugal sedimentation experiments with A4 and A7 suggests an antagonistic effect resulting from the mismatch between imidazoline-type lubricant II (L-5) and the fatty acid polyoxyethylene ester lubricant (L-2) component in the original sizing agent formula. The particle size d (0.9) of the remaining formulations showed no significant difference. When imidazoline-type lubricant III (L-6), saturated fatty acid polyoxyethylene ester lubricant (L-2), and amide-based cationic lubricant (L-3) were used in combination, the A1 sizing agent emulsion exhibited the smallest particle size d (0.9), measuring 0.721 µm.

#### 3.1.3. Emulsion Solid Content and Viscosity

**Figure 7:**
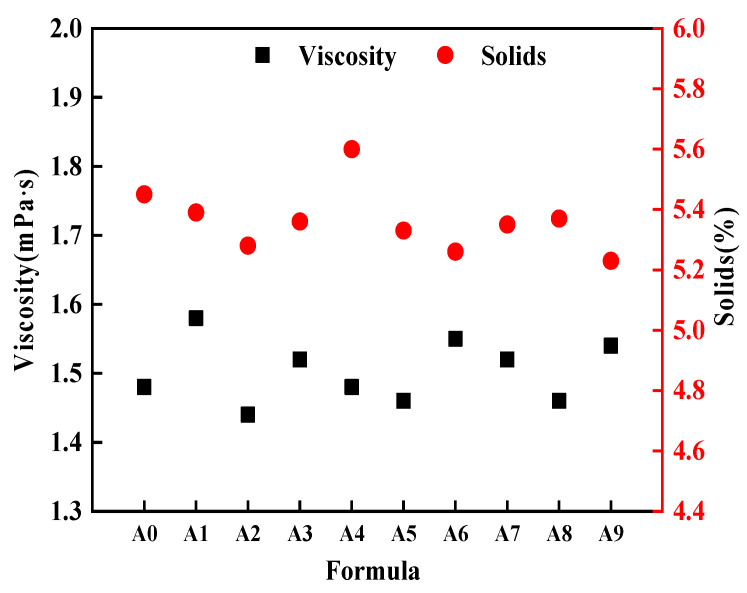
Solid content and viscosity of the sizing agent emulsion.

From Figure 7, it can be observed that the viscosity and solid content of different sizing agent formulations fluctuate within a certain range. The viscosity was generally in the range of 1.4~1.6 mPa, and the solid content was between 5 and 6%, with minor fluctuations. These values were well within the normal range for emulsions of continuous BF sizing agents. This indicated that the lubricant used in this study had a relatively minor impact on the sizing agent and solid content within the specified range. Moreover, there seems to be no inherent correlation between the solid content and viscosity of the sizing agent emulsion. However, it was noteworthy that formulations A2, A5, and A8, which contained the same imidazoline-type lubricant I (L-4), exhibited the lowest viscosity values. This indicates that L-4 can reduce the viscosity of the sizing agent emulsion to some extent. This is because the strong charge from the quaternary ammonium salt structure in L-4 molecules keeps them in a highly dispersed state within the solution. Through electrostatic repulsion, this charge prevents self-aggregation of L-4 molecules and the formation of over-crosslinked complexes with other components, thereby avoiding the creation of a high-viscosity network structure and reducing the system’s viscosity.

#### 3.1.4. Emulsion Surface Tension

**Figure 8:**
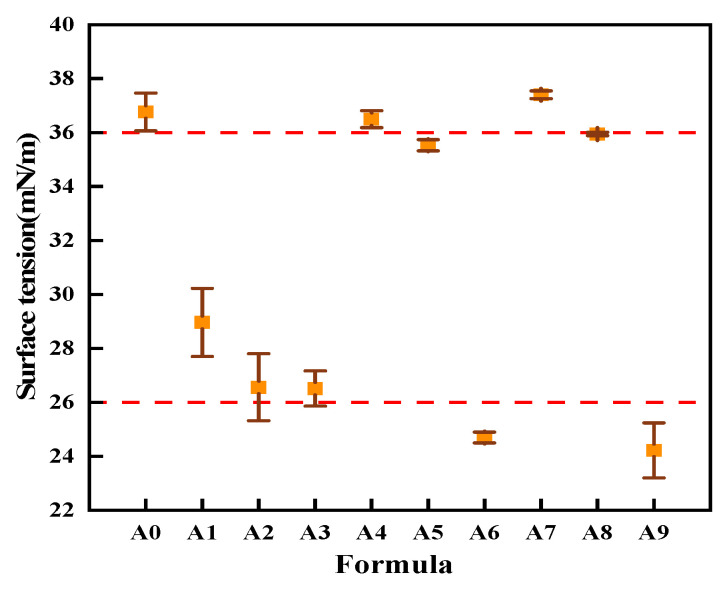
Surface tensions of emulsions with different sizing agents.

The surface tensions of the A0–A9 sizing agent emulsion formulations were tested, and the results are depicted in Figure 8. From the graph, it can be observed that there was a significant difference in the surface tension of emulsions with different formulations, and they exhibited a considerable fluctuation range (24~38 mN m^−1^). A phenomenon of “bipolarization” was evident, where the surface tensions of some sizing agent emulsions were concentrated around 26 mN m^−1^, while others were concentrated around 36 mN m^−1^. Notably, formulation A7 exhibited the highest surface tension (37.40 mN m^−1^), while formulation A9 showed the lowest (24.22 mN m^−1^). Through comparative analysis, it was discovered that when the original lubricants (L-1) and (L-3) remained unchanged, replacing lubricant (L-2) with lubricants L-4, L-5, and L-6 led to a significant reduction in the surface tension of the sizing agent emulsion. Among them, formulation A3 reached the lowest point, at 26.51 mN m^−1^. Additionally, substituting imidazoline-type lubricant III (L-6) for amide-based cationic lubricant (L-3, formulation A6) and non-ionic lubricant (L-1, formulation A9)resulted in a significant decrease in the surface tension of the sizing agent emulsion, reducing it to 24.69 mN m^−1^ and 24.22 mN m^−1^, respectively. Thus, it can be observed that imidazoline-type lubricant III (L-6) can significantly reduce the surface tension of the sizing agent emulsion.

### 3.2. Effect of Lubricants on BF Performances

#### 3.2.1. Basic Physical and Chemical Parameters

For a better comparative study of the influence of sizing agents on the performance of BF, an additional untreated fiber group was included as a blank control group (blank). The surface treatments of BF with various surfactant emulsions were evaluated for their effects on fiber linear density, diameter, moisture content, and combustible material content, as detailed in Table 2. From the table, it is evident that the type of lubricant has no significant effect on linear density, fiber diameter, moisture content, or combustible material content. In formulations A0~A9, the linear density was concentrated in the range of 225.32~255.57 tex, the moisture content ranged from 0.0697 to 0.0897%, and the combustible material content was 1.0446~2.1004% with a diameter of approximately 16 µm. Compared to the results with the untreated BF control group (blank), it was observed that both linear density and combustible material content increased while moisture content decreased. This was primarily attributed to the different components, such as film-forming agents, lubricants, and coupling agents, present in the A0 to A9 surfactant formulations, which ultimately affected the physical and chemical parameters of the fibers.

#### 3.2.2. Mechanical Performance

**Figure 9:**
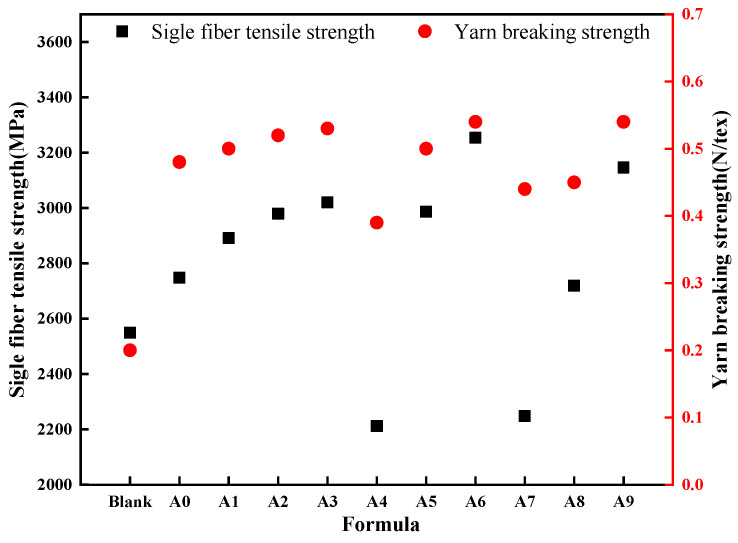
Mechanical performance of different BF.

The results obtained from the tensile strength test of single fibers and the yarn breaking strength for the A0–A9 sizing agent formulations in the emulsion drawing of BF are shown in Figure 9. As evident from the figure, compared to the blank group, the single fiber tensile strengths of BF obtained from the A4 and A7 formulations were lower, while the mechanical properties of BF from other formulations showed a significant improvement. The A3, A6, and A9 formulations utilized the imidazoline lubricant III (L-6), and they produced BF with single fiber tensile strengths and yarn breaking strengths of 3020.05 MPa, 3254.09 MPa, and 3146.05 MPa, and 0.53 N tex^−1^, 0.54 N tex^−1^, and 0.54 N tex^−1^, respectively. These values were significantly higher than those of other formulations. Among them, the A6 formulation produced BF with the best performance, with a single fiber tensile strength and yarn breaking strength 27.66% and 170.00% higher than the blank group, respectively. Compared to the original A0 formulation of BF, A6 exhibited an improvement of 18.42% and 12.50% in single fiber tensile strength and yarn breaking strength. This indicates that the lubricant combination utilizing imidazoline lubricant III (L-6) is beneficial for enhancing the mechanical properties of the fibers.

Due to the centrifugal settling treatment of sizing agent emulsions in formulations A4 and A7, the sediment remained after agitation or ultrasonic oscillation, indicating poor emulsion stability. Simultaneously, the large particle size of the sizing agent led to issues such as fiber fly and uneven coating of BF after treatment with these emulsions. Consequently, the mechanical properties of BF were compromised, and they failed to meet the requirements for industrialized drawing production of BF. Therefore, A4 and A7 were not the optimal formulations. The remaining seven formulations were further evaluated for enhancing the mechanical properties of Epoxy–BFRP using corresponding modified fiber fabrics.

### 3.3. Effect of Lubricants on Epoxy–BFRP Performances

#### 3.3.1. Mechanical Properties of Epoxy–BFRP

**Figure 10:**
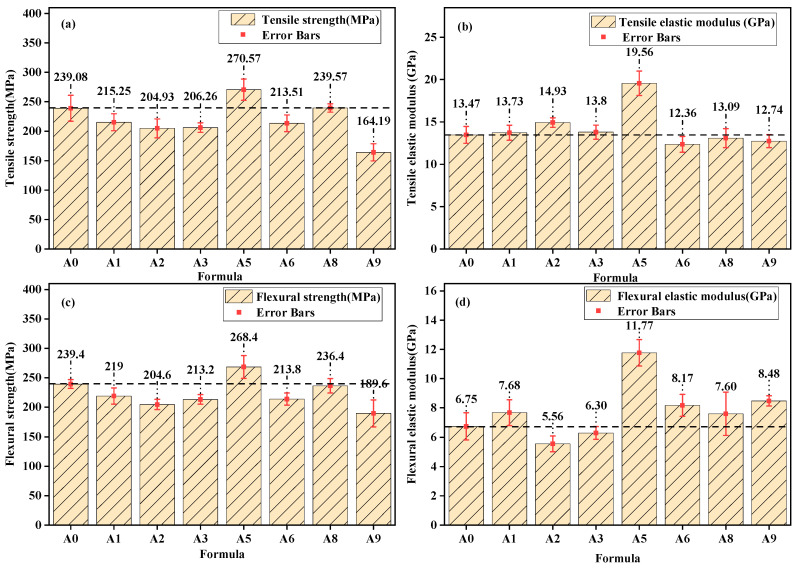
Mechanical properties of Epoxy–BFRP: Tensile strength (**a**), tensile modulus (**b**), flexural strength (**c**), flexural modulus (**d**).

Epoxy–BFRP was prepared using the seven selected formulations and the original sizing agent, and the corresponding tensile and flexural performances are shown in Figure 10. The results indicate that the mechanical properties of the Epoxy–BFRP obtained from the A5 formulation are superior to those of the A0 formulation. Significant improvements in mechanical performance were observed, with the highest tensile strength (270.57 MPa), flexural strength (268.4 MPa), and tensile modulus (19.56 GPa). Compared to the A0 formulation, the Epoxy–BFRP from the A5 formulation showed increases in tensile strength, tensile modulus, and flexural strength of 31.49 MPa (13.2%), 6.09 GPa (45.2%), and 29 MPa (12.0%), respectively. Although the mechanical properties of the BF obtained from the A1, A2, A3, A6, and A9 formulations were superior to those of the A0 formulation, the resulting tensile and flexural mechanical properties of Epoxy–BFRP did not surpass those of A0. Therefore, it was inferred that the mechanical properties of the fiber were not the key factors influencing the performance of Epoxy–BFRP. The permeability of the fiber to the resin matrix and the bonding ability between the fiber and the resin matrix were likely key factors affecting the performance of the composite material.

**Figure 11:**
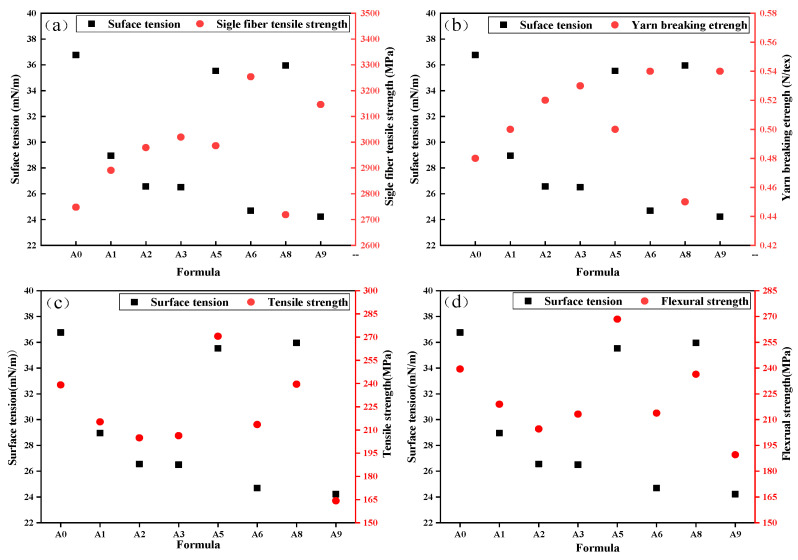
Correlation between surface tension of sizing agent emulsion and the mechanical properties of BF and Epoxy–BFRP: Surface tension and tensile strength of individual BF (**a**), surface tension and breaking strength of BF yarn (**b**), surface tension and tensile strength of Epoxy–BFRP (**c**), surface tension and flexural strength of Epoxy–BFRP (**d**).

In addition, the relationship between the surface tension of the sizing agent emulsion and the corresponding mechanical properties of BF and Epoxy–BFRP revealed a correlation, as illustrated in Figure 11. From the graph, it can be observed that the mechanical properties of BF and the surface tension of the sizing agent emulsion exhibited an overall negative correlation, while the mechanical properties of Epoxy–BFRP showed a positive correlation trend with the surface tension of the sizing agent emulsion. BF obtained from the A0, A5, and A8 formulations with a sizing agent emulsion surface tension of around 36 mN m^−1^ had relatively lower tensile strength and yarn breaking strength. However, the corresponding Epoxy–BFRP exhibited better mechanical properties. Additionally, the five formulations (A1, A2, A3, A6, and A9) with sizing agent emulsion surface tension below 30 mN m^−1^ resulted in significantly reduced mechanical properties of the corresponding Epoxy–BFRP. Among them, the three formulations (A3, A6, and A9) that contained imidazoline lubricant III (L-6) exhibited the highest single-fiber tensile strength and yarn breaking strength but had the poorest mechanical properties in the corresponding Epoxy–BFRP.

From the perspective of surface tension, the lower the surface tension of the sizing agent, the higher the spreading coefficient of the emulsion on the fiber surface and the smaller the solid–liquid contact angle, which enhances the spreading of the sizing agent on the fiber surface and affects fiber surface properties. However, a high dosage of sizing agent can instead hinder the contact between the resin and the fiber surface, further deteriorating the mechanical properties of the composite material—this is consistent with the experimental results in Figure 11. The reason for this phenomenon is that the interfacial compatibility during composite formation involves a three-phase synergistic interaction between the resin, emulsion, and fiber, rather than just two-phase interactions. Thus, the surface tension of the sizing agent must be controlled within a reasonable range: excessively low surface tension may impair the impregnation of fibers by the matrix resin and reduce bonding strength, thereby degrading the performance of Epoxy–BFRP.

#### 3.3.2. Morphology of Epoxy–BFRP

**Figure 12:**
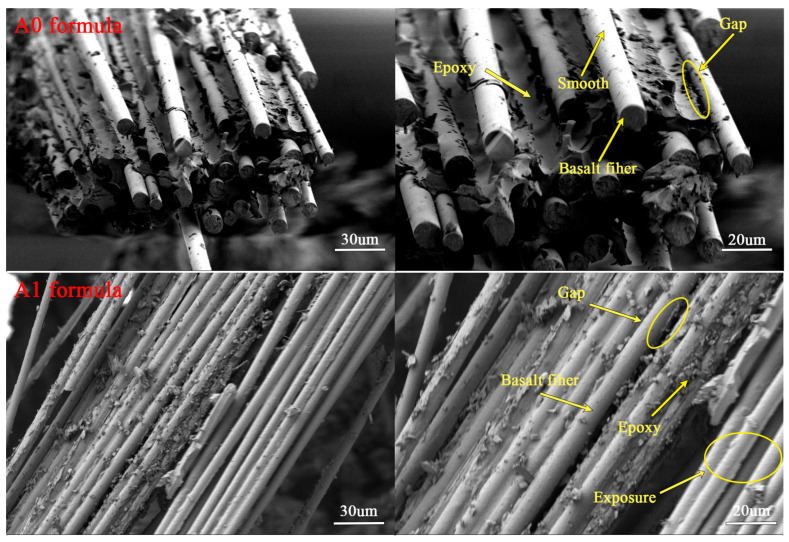

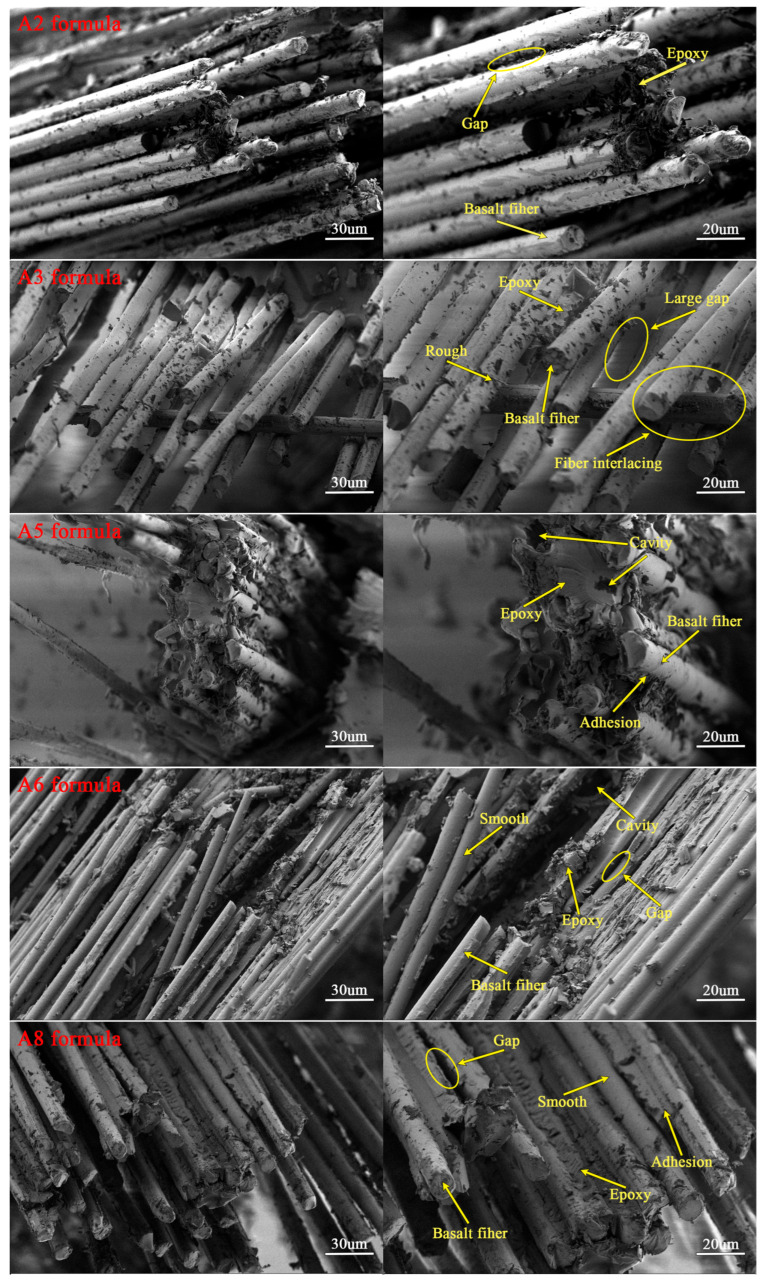

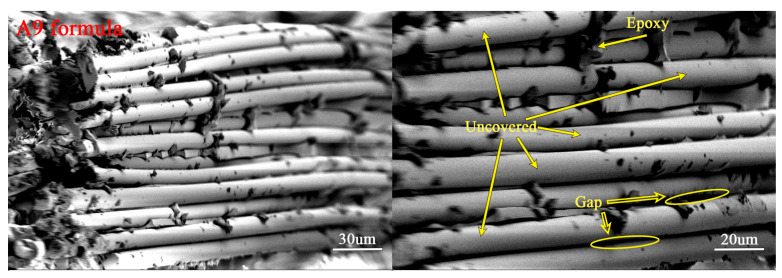
SEM Images of the flexural fracture surface of Epoxy–BFRP (left × 300, right × 500).

SEM images of the flexural fracture surfaces of eight types of Epoxy–BFRP are shown in Figure 12. From this figure, it can be observed that the SEM image of the flexural fracture of Epoxy–BFRP obtained from the A5 formulation did not exhibit obvious fiber–resin debonding phenomena. Gaps between fibers in the fracture surface were filled with resin, and only an extremely small amount of smooth-surfaced fibers can be seen. Additionally, some blocky resin was observed on the fiber surfaces. This visually confirms that the BF infiltrated with the A5 impregnating emulsion exhibited better bonding with the matrix resin, resulting in significantly higher flexural mechanical properties compared to other Epoxy–BFRPs. In comparison to the A5 formulation, the flexural fracture surface of Epoxy–BFRP obtained from the base formulation A0 also showed no apparent fiber–resin debonding, but a small number of smooth-surfaced fibers were present. Examination of the flexural fracture surface of Epoxy–BFRP from the A8 formulation revealed the presence of some smooth-surfaced fibers, along with clear instances of fiber–resin debonding. For Epoxy–BFRPs obtained from the A1, A2, A3, A6, and A9 formulations, their flexural fracture surfaces exhibited similar characteristics, with numerous smooth-surfaced fibers and visible fiber–matrix resin interface separation. In particular, the Epoxy–BFRP from the A9 formulation displayed a fracture surface dominated by exposed smooth fibers with little resin adhesion. This may indicate incomplete resin infiltration into the fibers, resulting in poor bonding at the fiber–resin interface. Under external forces, this leads to direct delamination at the interface, ultimately causing interface failure in the composite material, manifested as poor tensile and flexural strength.

## 4. Conclusions

Based on this study of the impact of lubricants in the sizing agent on the performance of BF and their Epoxy–BFRP, this work focused on the optimization of lubricant types and ratios. The physical and chemical parameters of the sizing agent and the properties of BF and Epoxy–BFRP obtained from different formulations were analyzed. The following conclusions were drawn:

(1) The lubricant components presented a significant influence on physical and chemical parameters of the sizing agent, as well as the properties of BF and its epoxy resin composite materials.
(2) Imidazoline-type lubricant II (L-5) exhibited antagonistic effects with lubricant L-2, leading to poor stability of the impregnating emulsion. Imidazoline-type lubricant III (L-6) can markedly reduce the surface tension of the sizing agent, yielding superior mechanical properties of the fibers.
(3) Imidazoline-type lubricant II (L-4) can partially reduce the viscosity of the impregnating emulsion, and the Epoxy–BFRP exhibited significant differences in the interface bonding performance between fibers and the matrix resin obtained by the A5 and A8 formulations. Specifically, for the A5 lubricant combination (L-1 0.30%, L-2 0.70%, L-4 0.30%), the tensile strength, tensile modulus, and flexural strength of the resulting Epoxy–BFRP increased by 13.2%, 45.2%, and 12%, respectively, which indicated excellent microscale interface properties.
(4) The particle size of the sizing agent emulsion can affect the mechanical properties of BF. A sizing agent emulsion with an excessively large particle size can lead to phenomena such as fly fibers and uneven coating of the BF after treatment, thereby degrading the mechanical properties of the BF.
(5) Epoxy–BFRP shows a positive correlation trend with the surface tension of the sizing agent emulsion.

## Figures and Tables

**Table 1:** The specific composition of lubricants in sizing agent emulsions (%).

Formula	Lubricant-I	Proportion	Lubricant-II	Proportion	Lubricant-III	Proportion
A0	L-1	0.30%	L-2	0.70%	L-3	0.30%
A1	L-1	0.30%	L-5	0.70%	L-3	0.30%
A2	L-1	0.30%	L-4	0.70%	L-3	0.30%
A3	L-1	0.30%	L-6	0.70%	L-3	0.30%
A4	L-1	0.30%	L-2	0.70%	L-5	0.30%
A5	L-1	0.30%	L-2	0.70%	L-4	0.30%
A6	L-1	0.30%	L-2	0.70%	L-6	0.30%
A7	L-5	0.30%	L-2	0.70%	L-3	0.30%
A8	L-4	0.30%	L-2	0.70%	L-3	0.30%
A9	L-6	0.30%	L-2	0.70%	L-3	0.30%

**Table 2:** Basic physical and chemical parameters of different BF.

Formula	Linear Density (tex)	Diameter (µm)	Moisture Content (%)	Combustible Content (%)
Blank	213.37	15.89	0.0913	0.9834
A0	243.53	15.73	0.0865	2.0030
A1	235.67	15.99	0.0697	1.0818
A2	225.32	15.88	0.0714	1.4393
A3	251.20	16.12	0.0784	1.0974
A4	247.93	16.98	0.0844	2.1004
A5	253.42	16.38	0.0765	1.0768
A6	236.64	15.56	0.0697	1.4348
A7	245.91	16.45	0.0897	1.0446
A8	255.57	16.88	0.0810	1.0768
A9	245.91	16.45	0.0897	2.0196

## Data Availability

The original contributions presented in the study are included in the article, further inquiries can be directed to the corresponding author.
